# Target-site and non-target-site based resistance to the herbicide tribenuron-methyl in flixweed (*Descurainia sophia* L.)

**DOI:** 10.1186/s12864-016-2915-8

**Published:** 2016-08-05

**Authors:** Qian Yang, Wei Deng, Xuefeng Li, Qin Yu, Lianyang Bai, Mingqi Zheng

**Affiliations:** 1 Department of Applied Chemistry, College of Science, China Agricultural University, Beijing, 100193 China; 2 Australian Herbicide Resistance Initiative, School of Plant Biology, University of Western Australia, Crawley, WA 6009 Australia; 3 Hunan Academy of Agricultural Science, Changsha, 410125 China

**Keywords:** Acetolactate synthase, Cytochrome P450, Flixweed, Tribenuron-methyl, Target-site based resistance, Non-target-site based resistance, Metabolic resistance, RNA-Seq

## Abstract

**Background:**

Flixweed (*Descurainia sophia* L.) is a troublesome and widespread broadleaf weed in winter fields in China, and has evolved high level resistance to acetolactate synthase (ALS)-inhibiting sulfonylurea herbicide tribenuron-methyl.

**Results:**

We identified a resistant flixweed population (N11) exhibiting 116.3-fold resistance to tribenuron-methyl relative to the susceptible population (SD8). Target-site ALS gene mutation Pro-197-Thr was identified in resistant plants. Moreover, the resistance can be reversed to 28.7-fold by the cytochrome P450 inhibitor malathion. The RNA-Sequencing was employed to identify candidate genes involved in non-target-site metabolic resistance in this population. Total 26 differentially expressed contigs were identified and eight of them (four P450s, one ABC transporter, three glycosyltransferase) verified by qRT-PCR. Consistent over-expression of the two contigs homology to CYP96A13 and ABCC1 transporter, respectively, were further qRT-PCR validated using additional plants from the resistant and susceptible populations.

**Conclusions:**

Tribenuron-methyl resistance in flixweed is controlled by target-site ALS mutation and non-target-site based mechanisms. Two genes, CYP96A13 and ABCC1 transporter, could play an important role in metabolic resistance to tribenuron-methyl in the resistant flixweed population and justify further functional studies.

**Electronic supplementary material:**

The online version of this article (doi:10.1186/s12864-016-2915-8) contains supplementary material, which is available to authorized users.

## Background

Herbicide resistance is the consequence of weed evolutionary adaptation to herbicide selection, and can be classified to target-site based resistance (TSR) and non-target-site based resistance (NTSR). TSR is widely reported in resistant weed species, which is endowed by gene mutations in target enzymes, such as acetolactate synthase (ALS)-, acetyl-CoA carboxylase (ACCase)-, protoporphyrinogen IX oxidase (PPO)-, 4-hydroxyphenylpyruvate dioxygenase (HPPD)-, and 5-enolpyruvylshikimate-3-phosphate synthase (EPSPS) [1–6]. To date, twenty-eight amino acid substitutions (numbers of amino acid in parentheses) in ALS endowing ALS herbicides resistance were identified at sites of Ala122 (3), Pro197 (13), Ala205 (2), Asp 376(1), Arg377 (1), Trp574 (3), Ser653 (3) and Gly654 (2) in weed species [5–7]. Non-target-site resistance is achieved by mechanisms reducing herbicide concentration reaching the target-site [1, 8]. One of the important NTSR mechanisms is the enhanced rates of herbicide metabolism (here in after referred to as metabolic resistance) often involving cytochrome P450 monooxygenase (thereafter referred to as P450), ABC transporter, glutathione S-transferase (GST), glycosyltransferase (GT) and peroxidase (POD) [9–12]. Compared to TSR, NTSR is less investigated (especially in broadleaf weed species) and remains poorly understood due to its complexity and diversity. NTSR may cause weeds evolve unpredictable resistance to diverse herbicides of different modes of action, even including herbicides not yet marketed [12, 13]. In addition, the resistance management strategies of herbicide mixtures and rotations, which are effective to manage TSR, may have little or no effects on NTSR metabolic resistance [12]. Therefore, NTSR threatens not only weed management, but also the utility of new herbicides.

Flixweed (*Descurainia sophia* L.) is a self-pollinated annual and notorious weed widely distributed in winter wheat cropping regions in China. Effective control of this weed heavily relied on the ALS-inhibiting herbicide (hereafter referred to as ALS herbicide) tribenuron-methyl, which targeted at ALS enzymes. Inhibition of ALS enzyme will affect synthesis of the branched-chain amino acids (Val, Leu and Ile) and eventually result in the death of plants. In addition, tribenuron-methyl can be absorbed by roots, stems, leaves, and transfer in weeds. Flixweed populations across the country have evolved high level resistance to tribenuron-methyl, and ALS gene mutation at Pro197 or Asp376 was found to decrease the enzyme sensitivity, which is mainly responsible for resistance to tribenuron-methyl in flixweed [14–19]. However, NTSR mechanisms endowing tribenuron-methyl-resistance in flixweed have not previously investigated.

RNA-Sequencing (RNA-Seq) has been recently used in transcriptome analysis of plant response to herbicide stresses in grass weeds *Eleusine indica* [20] and *Echinochloa cruss-galli* [21], and in identifying genes involved in NTSR in *Lolium rigidum* [22, 23] and *Alopecurus myosuroides* [24]. In the current study, a flixweed population with both TSR and NTSR mechanisms to tribenuron-methyl was identified. In particular, RNA transcriptome sequencing was conducted to identify genes involved in NTSR to tribenuron-methyl in this population. Two genes, CYP96A13 and ABCC1 transporter were deduced to play an important role in metabolic resistance to tribenuron-methyl in the resistant flixweed population. This is the first transcriptome-wide study in identifying NTSR genes in a broadleaf weed species.

## Results

To disclose the TSR and NTSR mechanisms to tribenuron-methyl, an R flixweed population N11 and an S population SD8 were used in this study. Tribenuron-methyl dose response, in vitro ALS activity and ALS gene sequencing were conducted to identify the TSR mechanism. The RNA-Seq was employed to identify candidate genes involved in NTSR in R population.

### Tribenuron-methyl dose response in the absence and presence of malathion

**Fig. 1:**
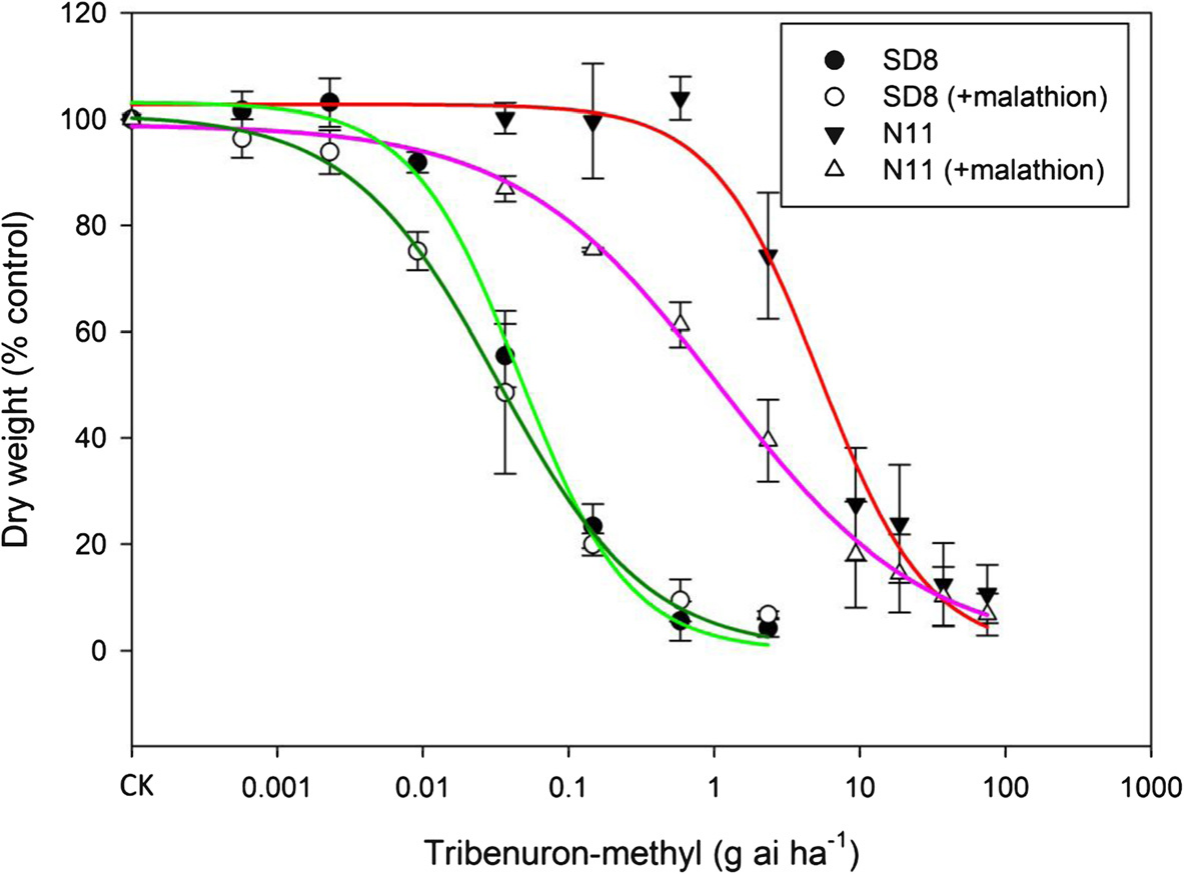
Dose–response curves of susceptible (SD8) and resistant (N11) flixweed populations to tribenuron-methyl in the absence and presence of cytochrome P450 inhibitor malathion. Each data point is the mean ± SE of two experiments

Whole-plant response experiments demonstrated that the R (N11) population has evolved a high level (116.3-fold) resistance to tribenuron-methly (Table 1). More importantly, the P450 inhibitor malathion can partially reverse the resistance (Fig. 1 and Table 1). Malathion alone at 720 g a.i. ha^−1^ had no visual effect on the growth of R and S plants. However, malathion greatly (4-fold) reduced the resistance level of the R (N11) population when used prior to tribenuron-methyl treatment. In contrast, malathion almost had no effects on the susceptibility of the S (SD8) population to tribenuron-methyl (Fig. 1 and Table 1). The P450 inhibitor malathion has long been used as a indicator of P450 involvement in metabolic resistance to ALS herbicides [25]. The results indicate that one or more P450s may mediate resistance to tribenuron-methyl in the R flixweed population.

**Table 1:** GR_50_ and I_50_ values of the susceptible (SD8) and resistant (N11) flixweed populations to tribenuron-methyl, in the absence and presence of cytochrome P450 inhibitor malathion

Herbicides	SD8 (S)		N11 (R)			
	GR_50_^a^	I_50_^b^	GR_50_^a^	RF^c^	I_50_^b^	RF^c^
Tribenuron-methyl	0.046a	1.77a	5.35b	116.3	54.65b	30.9
Tribenuron-methyl + malathion	0.039a	-	1.12c	28.7	-	-

Resistance levels were indicated by the resistance factor (RF). GR_50_ or I_50_ values with different letters are significantly different at P = 0.05 significant level. Data were means of two experiments

^a^ GR_50_, herbicide rate causing 50 % growth reduction of plants

^b^ I_50_, herbicide concentration causing 50 % inhibition of the ALS activity

^c^ RF (resistance factor) = GR_50_ or I_50_ (R)/GR_50_ or I_50_ (S)

### ALS activity assays in vitro

**Fig. 2:**
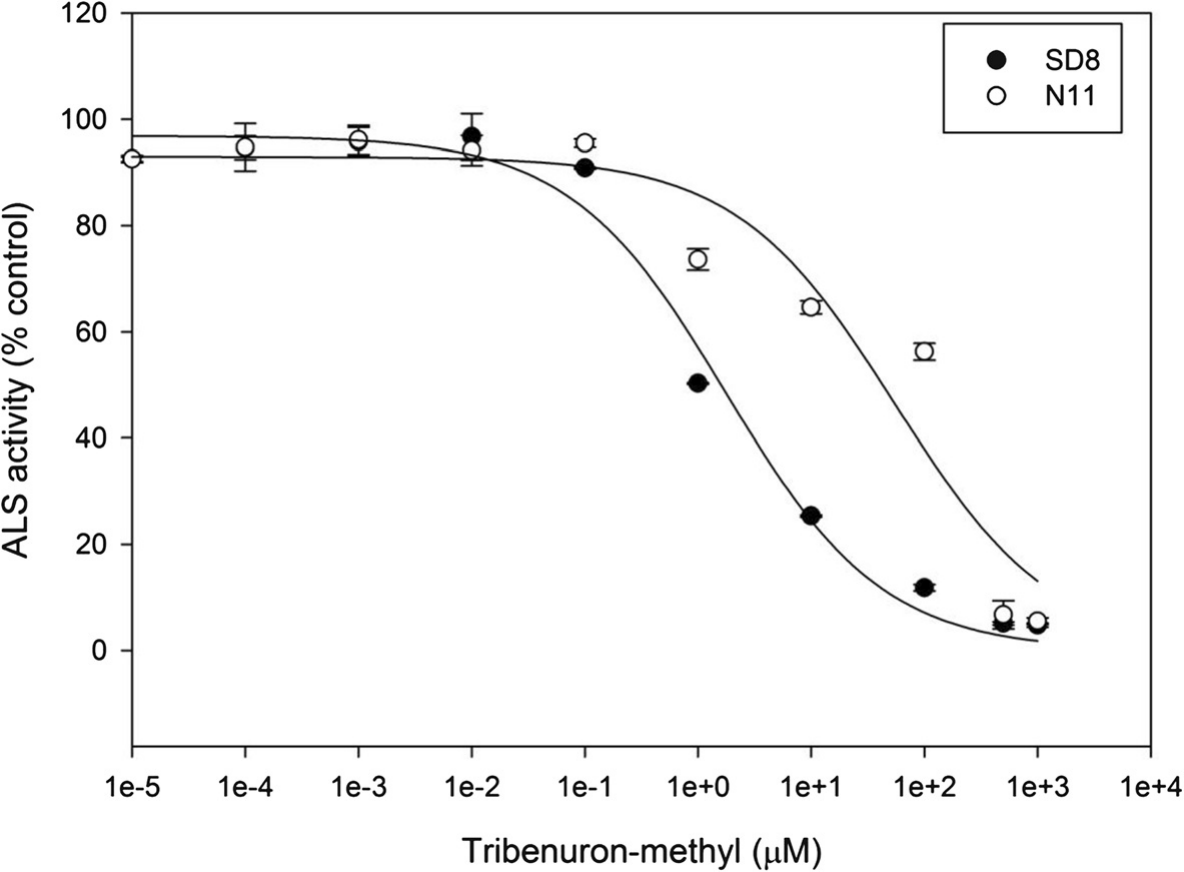
Effect of tribenuron-methyl on ALS activity of susceptible (SD8) and resistant (N11) flixweed populations. Each data point is the mean ± SE of two experiments

The ALS in vitro assay showed that ALS enzyme extracted from R (N11) plants was 30.9-fold resistant to tribenuron-methyl compared to that from S (SD8) plants (Table 1 and Fig. 2). The reduced sensitivity of ALS enzyme in R plants is likely due to mutation(s) in ALS gene.

### ALS gene sequencing

Two ALS genes with full lengths of 1998 bp and 2004 bp, respectively was cloned from S (SD8) and R (N11) plants. These two ALS genes displayed more than 95 % homology with the known ALS genes in flixweed (EMBL/Genbank accession JQ868736, JQ868737, JQ868738). A Pro-197-Thr substitution known to endow ALS herbicide resistance was identified in the 1998 bp ALS from R but not S plants. No amino acid substitutions were found in 2004 bp ALS from both R and S plants. It is clear that target-site ALS Pro-197-Thr mutation is in part responsible for tribenuron-methyl resistance in the R population.

### Transcriptome sequencing and assembly

RNA-Seq was conducted to identify genes involved in NTSR mechanism in resistant flixweed population. Total 33.36 Gb data with 272,121,520 raw reads were generated from the four libraries. After removing reads containing adaptor or ploy-N and low quality reads, there were 266,750,156 clean reads ranging from 31,286,811 to 36,457,368 per sample that were used for assembly (Additional file 1). Clean reads were assembled to 84,085 transcripts in the range of 201 to 15,697 bp. Among these transcripts, 52,898 unigenes > 200 bp, and 23,072 unigenes > 500 bp, with a mean length of 839 bp and an N50 length of 1,569 bp were obtained using the longest transcript in each loci of each gene (Table 2, Additional file 2).

**Table 2:** Summary of assembly quality for flixweed RNA-seq

Assembly quality parameters	
Total raw reads	272,121,520
Total clean reads	266,750,156
Clean bases	33.36G
Transcripts generated	84,085
No. unigenes > 200 bp	52,898
No. unigenes > 500 bp	23,072
Maximum length	15,697
Minimum length	201
Average unigene length	839
N50 value^a^	1569
N90 value^b^	305
Total nucleotides of unigenes	44,360,298

^a^ N50, 50 % of the assembled bases were incorporated into sequences with length of N50 or longer

^b^ N90, 90 % of the assembled bases were incorporated into sequences with length of N90 or longer

### Gene functional annotation and classification

Unigene annotation was performed by blast-searching against seven public databases. There were 35,211 (66.56 %) unigenes successfully annotated in at least one of NCBI non-redundant protein sequences database (Nr), NCBI non-redundant nucleotide sequences database (Nt), Pfam (Protein family), KOG/COG (Clusters of Orthologous Groups of proteins), Swiss-port, Kyoto Encyclopedia of Genes and Genomes (KEGG) and Gene ontology (GO) databases, with 5,118 (9.67 %) unigenes in all seven databases (Table 3). This includes 28,446 unigenes (53.77 %) that had a hit in Nr database, 30,571 (57.79 %) in Nt database and 21,179 (40.03 %) in Swiss-Prot database. The results of Nr database annotation indicate that the *D. sophia* has sequence similarities to *Arabidopsis lyrata* (28.5 % of total unigenes), *Arabidopsis thaliana* (26.4 %), *Capsella rubella* (20.8 %), *Eutrema salsugineum* (10.0 %) and *Brassica napus* (4.8 %) (Additional file 3).

**Table 3:** Gene annotation by BLAST-searching against public databases

Public database	Number of unigenes	Percentage (%)
Annotated in NR	28,446	53.77
Annotated in NT	30,571	57.79
Annotated in KEGG	9002	17.01
Annotated in SwissProt	21,179	40.03
Annotated in PFAM	19,032	35.97
Annotated in GO	21,670	40.96
Annotated in KOG	10,231	19.34
Annotated in all Databases	5118	9.67
Annotated in at least one Database	35,211	66.56
Total unigenes	52,898	100

**Fig. 3:**
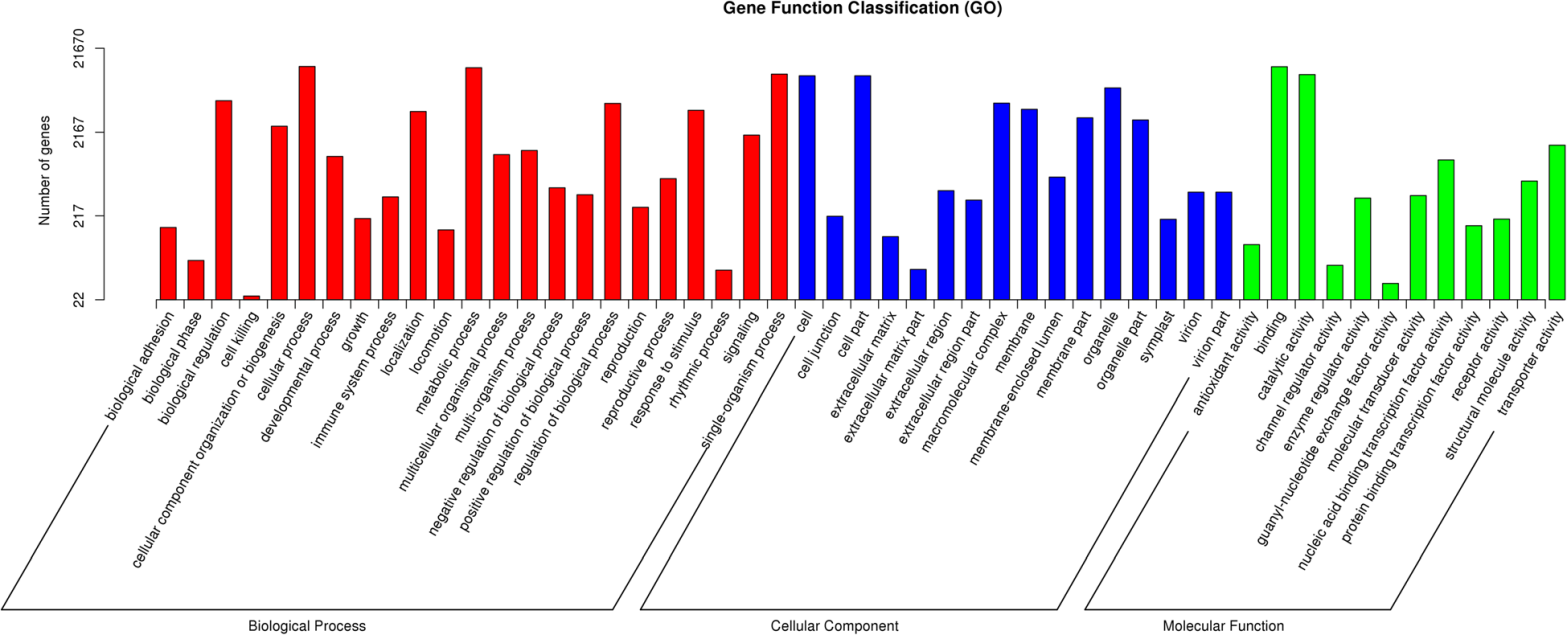
Gene ontology (GO) analysis of unigenes. The unigenes were summarized in biological process, cellular component and molecular function

GO assignments were used to predict the functions of unigenes by classifying them into ontology of molecular function (MF), biological process (BP) and cellular component (CC). There are 21,670 unigenes in R and S samples that were divided into 56 functional subgroups based on the sequence homologies. The largest gene subgroup in molecular function was binding (13,027 unigenes, 60.12 %), followed by catalytic activity (10,498 unigenes, 48.44 %) and transporter activity (1,517 unigenes, 7.00 %). The largest gene subgroup in biological process was cellular process (13,126 unigenes, 60.57 %), followed by metabolic process (12,669 unigenes, 58.46 %) and single-organism process (10,626 unigenes, 49.04 %). The largest gene subgroup in cellular component was cell (10,153 unigenes, 46.85 %), followed by cell part (10,152 unigenes, 46.85 %) and organelle (7,351 unigenes, 33.92 %) (Fig. 3).

**Fig. 4:**
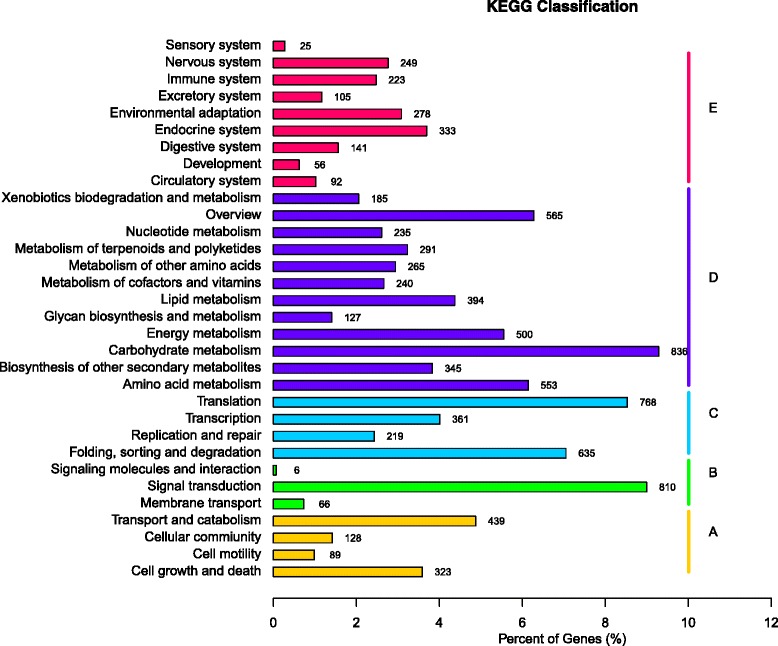
KEGG annotation of putative proteins. All 9,002 putative proteins showing significant homology to those in KEGG ortholog database were classified into 32 KEGG pathways. The y-axis represents KEGG pathways. The x-axis indicates number and percentage of a specific category of genes in each main classification. According to participation in KEGG pathways, genes were divided into five groups: A, cellular processes; B, environmental information processing; C, genetic information processing; D, metabolism; E, organism systems

KEGG is a platform for understanding functions and utilities of the gene products, especially large-scale molecular datasets generated by genome sequencing and other high-throughput experimental technologies. There are 9,002 unigenes that were classified into pathways of metabolism (4,536 unigenes, 50.39 %), genetic information processing (1,983 unigenes, 22.03 %), organismal systems (1,502 unigenes, 16.69 %), cellular processes (979 unigenes, 10.88 %) and environmental information processing (882 unigenes, 9.80 %) (Fig. 4) There are 883 unigenes that were annotated as NTSR-related genes including 258 P450s, 200 ABC transporters, 65 GSTs, 305 GTs and 55 PODs.

### Differential gene expression and functional enrichment analysis

In order to identify the candidate genes involved in resistance to tribenuron-methyl, differential expression analysis was performed using the DEGSeq program [26]. Pearson correlation coefficients (Additional file 4) demonstrated that the gene expression models among different plants within or between the two flixweed populations have high similarity. In total, 902 of 52,898 unigenes were identified to be significantly differentially expressed (q-value < 0.05 and |log_2_ (Fold change)| > 1) in the R and S plants, including 536 up- and 366 down-regulated in N11 (Additional file 5).

To further characterize the function of differentially expressed genes (DEGs), GO and KEGG enrichment analysis were conducted. In total, 574 DEGs were enriched in 2,323 GO terms, with 171 DEGs significantly enriched in 9 GO terms. We used up-regulated and down-regulated DEGs to perform GO enrichment analysis, respectively. Among the up-regulated genes, three GO terms including “response to UV-B” (7) and “response to UV” (8) in the category of BP; “transition metal ion binding” (63) in the category of MF were significantly enriched between R (N11) and S (SD8) samples (Additional file 6-A). Among the down-regulated genes, GO terms including “DNA integration” (9), “glycerol metabolic process” (5) and “alditol metabolic process” (5) in the category of BP; “ATPase activity, coupled to transmembrane movement of ions, phosphorylative mechanism” (6) and “cysteine-type peptidase activity” (10) in the category of MF were significantly enriched between N11 and SD8 samples (Additional file 6-B).

Total 295 genes (165 up- *vs* 130 down-regulated in the R plants) were enriched into 113 KEGG pathways (Addditional file 7). The top-five enriched pathways were metabolism of xenobiotics by P450 (8), drug metabolising-P450 (8), retinol metabolism (5), naphthalene degradation (4) and degradation of aromatic compounds (4) (Table 4). In the KEGG pathway analysis, genes involved in “metabolism by P450” changed significantly, suggesting P450 genes may play a vital role in metabolic resistance to tribenuron-methyl in flixweed.

**Table 4:** The top-ten enriched KEGG pathway terms in DEGs between N11 and SD8

Term^a^	ID^b^	Sample number^c^	Background number^d^	Corrected *P*-Value
Metabolism of xenobiotics by P450	ko00980	8	54	6.07 × 10^−4^
Drug metabolism - cytochrome P450	ko00982	8	54	6.07 × 10^−4^
Retinol metabolism	ko00830	5	18	1.45 × 10^−3^
Naphthalene degradation	ko00626	4	12	3.79 × 10^−3^
Degradation of aromatic compounds	ko01220	4	13	3.91 × 10^−3^
Tryptophan metabolism	ko00380	6	47	4.70 × 10^−3^
Tyrosine metabolism	ko00350	6	47	4.70 × 10^−3^
Chloroalkane and chloroalkene degradation	ko00625	4	17	5.82 × 10^−3^
Nitrogen metabolism	ko00910	5	41	1.42 × 10^−2^
Phenylpropanoid biosynthesis	ko00940	9	148	1.85 × 10^−2^

^a^ description of KEGG pathway

^b^ numbered information of unique pathway in KEGG database

^c^ number of DEGs enriched in this pathway

^d^ number of unigenes annotated in this pathway

### Differential expressed contigs involved in NTSR

Among 536 up-regulated contigs in R (N11), no contigs were annotated to the target ALS enzyme. There are 37 contigs (26 up- and 11 down-regulated in R) annotated as metabolism enzymes including 11 P450s, 3 ABC Transporters, 5 GSTs, 16 GTs and 2 PODs. In addition, there are 65 up-regulated contigs in N11 which were not annotated by GO or KEGG enrichment.

Given the important roles of metabolic enzymes in herbicide metabolism and resistance, the contigs that were up-regulated in R samples (N11) and that annotated as metabolic enzymes were selected as the candidate metabolic resistance contigs. Total 26 contigs were selected as candidate genes that may confer tribenuron-methyl resistance. Of which, eight contigs were annotated to P450 families, two annotated to ABC transporter families, two annotated to GST families, thirteen annotated to UGT families and one annotated to POD (Table 5).

**Table 5:** qRT-PCR validations with metabolic resistance candidate genes in flixweed

Gene ID	Function annotation	Padj^a^	FPKM ^b^ (R/S)	Fold change: qRT-PCR validation (2^-△Ct^)	
				RNA-Seq samples	Addtional samples
c29425_g1	Cyt P450, CYP77B1/77A3	1.31 × 10^−15^	69.5	33.88*	0.57
c1867_g1	CytP450, CYP96A13	2.55 × 10^−10^	24.94	16.55**	11.94**
c21381_g1	Cyt P450, CYP71A3	4. 25 × 10^−3^	22.63	4.06	1.28
c22393_g1	Cyt P450, CYP86B1	9.35 × 10^−11^	20.82	4.24*	2.75*
c17043_g1	CytP450, CYP96A15	2.28 × 10^−6^	16.58	448.6***	24.76*
c18590_g1	CytP450, CYP71B14	3.9 × 10^−2^	12.87	1.82	1.3
c17358_g1	CytP450, CYP734A1	8.39 × 10^−3^	2.91	1.95	0.85
c25953_g1	Cyt P450, CYP81F1/81 F3	4.33 × 10^−2^	2.64	2.69	0.27
c11932_g1	ABC transporter, ABCC1	3.42 × 10^−12^	Inf ^c^	153.43*	9.26***
c31018_g1	ABC transporter, ABCF5	1.82 × 10^−2^	2.46	1.21	0.6
c25791_g1	GST, F11	2.46 × 10^−3^	3.11	1.24	1.56
c21822_g2	GST, U17	7.10 × 10^−3^	2.87	1.46	0.86
c19559_g2	UDP-glycosyltransferase 78D4	1.06 × 10^−3^	14.87	2.42	0.94
c33777_g2	UDP-glycosyltransferase 91A1	4.03 × 10^−3^	6.52	2.36	0.74
c24861_g1	UDP-glycosyltransferase 84A1	1.33 × 10^−3^	5.76	5.33*	0.98
c12944_g1	UDP-glycosyltransferase 71B1	3.02 × 10^−6^	5.01	2.60	0.5
c31999_g1	UDP-glycosyltransferase 71C2	8.92 × 10^−5^	4.49	1.98	0.67
c5029_g1	UDP-glycosyltransferase 79B6	9.54 × 10^−5^	4.15	3.62	0.54
c15368_g1	UDP-glycosyltransferase 91A1	4.68 × 10^−3^	3.71	2.91	0.7
c19559_g1	UDP-glycosyltransferase 78D4	2.38 × 10^−2^	3.58	4.82*	0.89
c33777_g1	UDP-glycosyltransferase 91A1	1.53 × 10^−3^	3.08	3.16*	0.53
c29603_g1	Glycosyl transferases group 1	1.64 × 10^−2^	2.67	1.86	0.92
c30538_g1	Glycosyl transferase family 21	3.56 × 10^−2^	2.56	2.03	0.9
c31908_g1	Glycosyl transferase family 21	2.28 × 10^−2^	2.51	1.46	0.78
c29566_g1	UDP-glycosyltransferase 74D1	4.07 × 10^−2^	2.51	1.65	1.18
c28527_g1	peroxidase activity	2.89 × 10^−2^	4.01	2.15	1.14

Identification of differentially expressed contigs between tribenuron-methyl susceptible (SD8) and resistant (N11) flixweed populations by RNA-Seq, and the expression levels of the contigs were validated using qRT-PCR with RNA-Seq samples (*n* = 2) and additional samples from resistant N11 (*n* = 12) relative to susceptible SD8 (*n* = 11). Fold-change in gene expression (2^-△Ct^) was calculated relative to the susceptible samples, where △C_T_ = [C_T_ target gene-C_T_ mean of two internal control genes]. *P*-value of <0.05, 0.01, 0.001 is indicated by *, **, and ***, respectively (from SPSS analysis)

^a^ Padj, adjusted *P*-value, the differentially expressed contigs were fliterred by Padj < 0.05 from DESeq analysis

^b^ FPKM, fragments per kilobase of transcript sequence per millions base pairs sequenced

^c^ Inf indicates readcount of SD8 samples = 0 in RNA-Seq

### qRT-PCR validation of candidate NTSR gene expression

The expression of 26 candidate contigs in RNA-seq samples were validated by qRT-PCR. The specificity of each primer pairs designed on the basis of unigene sequences was verified by amplicon sequencing. Two internal control genes (18 s rRNA and ALS) were used in qRT-PCR and the results were normalized as 2^-△Ct^ using the method of Schmittgen and Livak [27]. The results (Table 5) showed that 8 of 26 candidate contigs exhibited significantly higher expression levels in the R (N11) than in S samples (SD8). These 8 contigs include four P450s, one ABC transporter and three GTs.

The expression patterns of the 8 candidate contigs were also examined in 11 additional plants from the S (SD8) and 12 from the R population (N11) respectively. The S and R plants used for qRT-PCR were grown under the same conditions, and RNAs extracted at the same time as the plants for RNA-Seq. Based on the results of RNA-Seq and qRT-PCR validation (Table 5), 4 out of 26 candidate contigs were expressed, on average, significantly higher in R (N11) than in S (SD8) plants examined. These include three P450s contigs (c1867_g1, c22393_g1, c17043_g1) homology to CYP96A13, CYP86B1 and CYP96A15, respectively, and a contig (c11932_g1) annotated as an ABC transporter ABCC1. However, there are variations in individual plants in their expression (data not shown). For example, expressions of contigs c17043_g1 and c22393_g1, which annotated as CYP96A15 and CYP86B1, were up-regulated only in 8 and 5 of 12 individual plants, respectively. Therefore, only contigs c1867_g1 (CYP96A13) and c11932_g1 (ABCC1) were consistently and significantly over-expressed in each R individuals.

## Discussion

In this study we demonstrate that resistance to tribenuron-methyl in the R population (N11) is due to target-site ALS Pro-197-Thr mutation plus non-target-site resistance mechanisms because (1) the known Pro-197-Thr mutation only provides partial (31-fold) resistance whereas at the whole plant level a more than 100-fold resistance was observed (Table 1), and (2) a known P450 inhibitor malathion can largely reverse the resistance (Table 1 and Fig. 1). It has been well established that malathion can inhibit sulfonylurea herbicide metabolism, thus reversing metabolism-based resistance [1, 12]. Metabolism-based resistance to ALS and ACCase-inhibiting herbicides has long been reported and are increasing in both monocot and dicots weed species [5, 28]. However, identification of herbicide-metabolizing and resistance-endowing genes has been slow and only recently a few P450 genes have been identified in grass weed species [29]. In particular, RNA-Seq has been successfully used to identify genes involved in metabolic resistance to ALS herbicides in two grass weed species *L. rigidum* [23] and *A. myosuroides* [24]. However, studies on metabolic herbicide resistance in broadleaf weed species are limited and genes remain unknown. Flixweed is a broad leaf, economically important weed species in China and many populations have evolved high level resistance to the ALS herbicides tribenuron-methyl. Identifying genes involved in NTSR are important for understanding metabolic resistance evolution in this dicot weed species and for development of resistance mitigation strategies.

In this study, 272,121,520 raw reads and 266,750,156 clean reads were generated in flixweed by the Illumina Hiseq 2500 technology, enabling assembling of 84,085 transcripts and 52,898 unigenes (Table 2). The N50 size value of unigenes was 1,569 bp which is comparable or longer than those obtained in recent *de novo* weed transcriptome assemblies based on Illumina [20, 23, 24]. Because the RNA-seq dataset was obtained from the above-ground material of only four 40 day-old individuals, it may not cover high numbers of total unigenes. However, differential expressed genes in xenobiotic and drug metabolism pathways involving P450 were enriched and well represented (Additional file 7). Although there were only two biological replicates for each population, the gene expression patterns in the four samples exhibited high similarity (Additional file 4). In addition, in this study, we primarily focused on those defensive genes that constitutively differently expressed between the R and S plants, which we consider to be more effective and efficient in endowing resistance than those genes induced after herbicide treatment.

Among the 536 up-regulated contigs, 26 contigs were annotated to metabolism enzymes including P450, ABC transporter, GST, GT and POD. Of the 26 contigs, eight contigs were validated by qRT-PCR in RNA-seq samples and selected as candidate NTSR genes (Table 5). The eight candidate NTSR genes identified here potentially encoded proteins with homology to four P450s (CYP77A3/CYP77B1, CYP96A13, CYP86B1, CYP96A15), one ABC transporter (ABCC1) and three GTs (UGT84A1, UGT78D4, UGT91A1).

Further qRT-PCR validation with additional samples from the R and S populations identified 4 contigs that expressed on average significantly higher in R than in S plants (Table 5). While expression of CYP96A13 and ABCC1 was consistently and significantly higher in all individual R plants than that in all S plants tested, expression of CYP86B1 and CYP96A15 were not universally higher in all R individuals. This indicates that CYP96A13 and ABCC1 are highly associated with tribenuron-methyl resistance, and CYP86B1 and CYP96A15 need further confirmation. Nevertheless, expression variations among individual plants of validated NTSR-related genes had previously been observed in *L. rigidum* and *E. phyllopogon* [22, 23, 29]. There are possibilities that individual plants may use slightly different sets of resistance genes to achieve different levels of resistance.

P450s identified so far in ALS tolerant crops or resistant weeds include CYP76B1 in *H. tuberosus* metabolizing phenylurea herbicide [30]; CYP71C6v1 in wheat metabolising sulfonylurea herbicides chlorsulfuron and triasulfuron [31]; CYP76C subfamily in *A. thaliana* metabolizing monoterpenol [32]; CYP72A31 and CYP81A6 in *O. sativa* or *A. thaliana* metabolizing bensulfuron-methyl (BSM) [33–35]; CYP81A12 and CYP81A21 in *E. phyllopogon* metabolizing bensulfuron-methyl (BSM) and penoxsulam (PX) [29]. However, there is lack of reports on involvement of the three candidate P450s (CYP96A13, CYP86B1 and CYP96A15) in herbicide metabolism and resistance. This is likely due to (1) difference in P450s involved in ALS herbicide metabolism in grass and broadleaf species, (2) difference in P450s in metabolizing specific ALS herbicides, and (3) insufficient studies.

Notwithstanding this, these P450s have been reported to play important roles in metabolism of secondary metabolites and catalyze diverse reactions in plants. For example, CYP96A15 was reported to catalyze the hydroxylation of alkane substrate, and involve in biosynthesis of wax secondary alcohols and ketones [36]. CYP86A and CYP86B subfamilies catalyze ɷ-hydroxylation of fatty acids, and involve in the biosynthesis of cutin or suberin in plants [37, 38]. The hydroxylation and epoxidation catalyzed by P450s are also the important degradation pathways for herbicides [39, 40]. Hence, the three candidate P450s may play important roles in tribenuron-methyl metabolism and resistance in *D. sophia*. Although correlation of the three P450 candidate genes and tribenuron-methyl resistance was further validated in additionally 11 S (SD8) and 12 R (N11) samples, more samples are needed to understand the complexity at the population level. For instance, if all the resistant plants employ the same or different sets of P450s in resistance? In cross-pollinated grass weed species (e.g., *L. rigidum* and *A. myosuroides*), metabolic herbicide resistance is likely more complex as different individuals may use different sets of candidate P450s [22, 24]. The next step is functional characterization of these candidate P450s genes using yeast or plant transgenic systems.

In higher plants, ATP-binding cassette (ABC) transporters have been implicated in detoxification of xenobiotic including herbicides [41, 42]. In contrast to the P450s which detoxify herbicides by metabolism, ABC transporters detoxify herbicides and confer herbicide resistance by compartmentation of herbicides and their metabolites [42]. In this current study, the contig (c11932_g1) was annotated to ABC transporter ABCC1 and associate with tribenuron-methyl resistance. The vacuolar Arabidopsis multidrug resistance-associated protein 1 (*At*ABCC1/*At*MRP1) was the first plant ABC transporters to be studied in depth, which transports several glutathione conjugates including the chloroacetanilide herbicide, metolachlor [43]. Although there is no direct evidence on involvement of ABCC1 in herbicide resistance in weeds, considerable evidence in vitro and in vivo experimental evidence suggests that MRP1 (ABCC1) has a role in protecting tissues from toxin induced damage [44]. Two ABCC-type transporters (*At*ABCC1 and *At*ABCC2) in *A. thaliana* were reported to mediate tolerance of arsenic and arsenic-based herbicides [45]. Over expression of *At*Pgp1, a multi-drug resistant family member, and its garden pea homolog psNTP9 have been shown to confer multi-herbicide resistance in *A. thaliana* [46]. In addition, the *At*MRP4 (homologues to *Ta*MRP1) was reported to be induced by the herbicide safer cloquintocet-mexyl to protect wheat from clodinafop-propargyl injury by transporting herbicide-GST conjugates to vacuoles [47]. Therefore, it is likely that ABC transporter ABCC1 is involved in compartmentation of tribenuron-methyl metabolites in resistant flixweed.

GST and GT are two important herbicide-detoxifying enzymes and play important roles in metabolism and NTSR to herbicides [48–50]. It is noticed that GSTs and GTs in *L. rigidum* were associated with NTSR to the ACCase and ALS herbicide [22, 23]. In this study, two GST and 13 GT contigs were annotated respectively, nevertheless, the average expression levels of these GST- and GT-annotated contigs showed no significant differences between the R and S plants in validation experiments with additional plants (Table 5). POD is another metabolic enzyme and plays important roles in plant defense [51]. Whereas, there is few report on its involving herbicide metabolism. In this study, one contig was annotated to POD. Moreover, the expression level of this contig exhibited no significant difference between S and R plants in validation experiments (Table 5). Hence, these GSTs, GTs and PODs unlikely play a role in tribenuron-methyl resistance in flixweed.

## Conclusions

A flixweed population (N11) highly resistant to the ALS herbicide tribenuron-methyl was identified, displaying both TSR and NTSR mechanisms. The TSR is due to a known resistance-conferring ALS mutation of Pro-197-Thr and the NTSR is likely due to P450-mediated metabolic resistance and ABC transporter- mediated sequestration of the metabolites. The c1867_g1 (homology to CYP96A13) and c11932_g1 (homology to ABCC1) contigs are potential NTSR genes or markers, for metabolic resistance to tribenuron-methyl in flixweed. Functional characterization of the identified P450 gene (CYP96A13) and the transporter gene (ABCC1) using transgenic plants is therefore warranted. This study will greatly extend our understanding the herbicide resistance mechanisms, and may identify the genes involving in herbicide metabolism.

## Methods

### Plants materials

Mature seeds of the susceptible (S) flixweed population (SD8) were collected from roadsides at Linyi in Shandong province (N35°05’45.00”, E118°09’3.78”) that had never been treated with herbicides. Seeds of the resistant (R) flixweed population (N11), were harvested randomly from winter wheat fields at Baoding of Hebei province (N38°36’32.80”, E115°01’52.50”) in 2013.

Flixweed seeds were polished by abrasive paper for 30s, and then immersed in 0.3 % gibberellin solution for 30 min. After rinsing thoroughly with distilled water, they were germinated in Petri dishes for 72 h. Germinating seedlings were transplanted into 9-cm diameter plastic pots containing moist loam soil, and then kept in a climate chamber at 20 °C/15 °C (day/night), 14 h photoperiod with light intensity of 20,000 Lux [16].

### Tribenuron-methyl dose response in the absence and presence of the cytochrome P450 inhibitor malathion

Whole-plant response experiments were conducted to determine the sensitivities of SD8 and N11 populations to tribenuron-methyl in the absence and presence of malathion. Malathion at the rate of 720 g a.i.ha^−1^ has no visual effects on flixweed seedling growth, and therefore, was used to treat the plants 30 min prior to tribenuron-methyl treatment. Tribenuron-methyl (diluted in water containing 0.3 % Tween-80) rates of 5.72 × 10^−4^, 2.29 × 10^−3^, 9.16 × 10^−3^, 3.66 × 10^−2^, 0.15, 0.59, 2.34 g a.i.ha^−1^ were used to treat the S plants, and 3.66×10^−2^, 0.15, 0.59, 2.34, 9.38, 18.75, 37.50, 75 g a.i.ha^−1^ for the R plants. Control plants were treated with 0.3 % Tween-80 solution.

Tribenuron-methyl was applied using a moving-boom cabinet sprayer delivering 600 L ha^−1^ water at a pressure of 0.4 MPa by a flat fan nozzle positioned at 54 cm above the foliage. Treated plants were returned to the climate chamber. The aboveground shoots were harvested 21 days after treatment and the dry weight determined. The experiment was conducted with three replicates per herbicide dose and repeated once.

### In vitro ALS activity

The 5-6 leaf stage seedlings (40 days after transplant) were used for in vitro ALS activity assay. The ALS extraction and activity assay were conducted according to the methods described by Yu et al. [52] and Deng et al. [17]. ALS activity was determined colorimetrically (520 nm) with microplate photometer (Thermo Fisher) by measuring acetoin production. Each assay was conducted with three replicates and repeated two times with independent enzyme extracts. Technical grade tribenuron-methyl was used in the reaction mixture with the final concentrations of 1.0×10^−5^, 1.0×10^−4^, 1.0×10^−3^, 1.0×10^−2^, 0.1, 1.0, 10, 1.0×10^2^, 5.0×10^2^ and 1.0×10^3^ μM, respectively.

### Statistical analyses

Data obtained from tribenuron-methyl dose–response and in vitro ALS activity assay were converted into percentage of the control and subjected to the non-linear regression analysis. The herbicide rates causing 50 % plant growth reduction (GR_50_) and herbicide concentration causing 50 % inhibition of ALS activity (I_50_) were evaluated using the four-parameter log-logistic Eq. proposed by Seefeldt et al. [53].

$$ y=C + \left(D\mathit{\hbox{--}}C\right)\ /\ \left[1 + {\left(x/\ {\mathrm{I}}_{50}\mathrm{or}\ \mathrm{G}{\mathrm{R}}_{50}\right)}^b\right] $$

Where *C* is the lower limit, *D* is the upper limit, *b* is the slope at the I_50_ or GR_50_. The resistance factor (RF) was calculated by the GR_50_ or I_50_ of the R population divided by that of the S population to estimate the resistance levels.

### ALS gene sequencing

Genomic DNA was extracted from shoot tissue of the four-leaf stage individual plants using the DNA extraction kit (Tiangen, Beijing, China). Primers (forward: 5′-TCTATCTCTCGCTCCTCTCC-3′; reverse: 5′-GCGTCTGAAGTAAATGAAAAAC-3′) were designed based on the ALS sequences of flixweed (FJ715633). The 25 μL polymerase chain reaction (PCR) mixture consisted of 100 ng of genomic DNA, 0.5 μL (25 μM) of each primer and 12.5 μL of 2 × Taq PCR Master Mix and 10.5 μL of ddH_2_O (Tiangen, Beijing, China). PCR was run with the following program: denaturation at 94 °C for 3 min, 35 cycles of 94 °C for 45 s, 58 °C for 45 s and 72 °C for 2 min 30 s, followed finally by an extension step of 10 min at 72 °C. PCR product was purified from agarose gel with TIAN gel Midi Purification Kit (Tiangen, Beijing, China).

The purified PCR products were cloned into the pGM-T vector and transformed into TOP10 Chemically Competent Cells. *Escherichia coli* clones containing the insert were sequenced directly with the universal primers T7 and SP6 commercially (Sangon, Shanghai, China). Eight clones of each plant (*n* = 10) were sequenced respectively for S (SD8) and R (N11) populations. ALS sequences of S and R plants were compared with the published susceptible sequence (BJ25) (EMBL/Genbank accession JQ868736) to determine the potential resistance mutations.

### RNA extraction and quality determination for RNA-Seq

Total RNA was extracted from leaves of two individual R and S plants, respectively, using the RNApre Pure Plant Kit (Tiangen, Beijing, China). RNA purity was checked using the NanoPhotometer® spectrophotometer (IMPLEN, CA, USA), and RNA concentration were determined using the Qubit® RNA Assay Kit (Qubit®2.0 Flurometer, Life Technologies, CA, USA). RNA integrity was assessed using the RNA Nano 6000 Assay Kit (Agilent Bioanalyzer 2100 system, Agilent Technologies, USA). The best qualified RNA samples were chosen for cDNA library preparation.

### cDNA library construction and sequencing

A total amount of 3 μg RNA per sample was used as input material for the RNA sample preparations. Sequencing libraries were generated using the NEBNext® Ultra™ RNA Library Prep Kit for Illumina® (NEB, USA) following manufacturer’s recommendations, and mRNA purified from total RNA using poly-T oligo-linked magnetic beads. Fragmentation was carried out using divalent cations under elevated temperature in NEBNext first strand synthesis reaction buffer (5X). First strand cDNA was synthesized using random hexamer primer and M-MuLV reverse transcriptase (RNaseH). Second strand cDNA synthesis was subsequently performed using DNA polymerase I and RNase H. Remaining overhangs were converted into blunt ends via exonuclease/polymerase activities. After adenylation of 3’ ends of DNA fragments, NEBNext adaptors with hairpin loop structure were ligated to prepare for hybridization. In order to select cDNA fragments of preferentially 150 ~ 200 bp in length, the library fragments were purified with the AMPure XP system (Beckman Coulter, Beverly, USA). Then 3 μL USER Enzyme (NEB, USA) was used with size-selected, adaptor-ligated cDNA at 37 °C for 15 min followed by 5 min at 95 °C before PCR. Then PCR was performed with Phusion High-Fidelity DNA polymerase, universal PCR primers and index (X) primers. Finally, PCR products were purified (AMPure XP system) and library quality assessed on the Agilent Bioanalyzer 2100 system. The clustering of the index-coded samples was performed on a cBot Cluster Generation System using the TruSeq PE Cluster Kit v3-cBot-HS (Illumina). After cluster generation, four libraries (N11_4, N11_5, SD8_3 and SD8_4) preparations were sequenced on the Illumina Hiseq 2500 platform and 125 bp paired-end reads were generated.

### Transcriptome assembly and gene functional annotation

Clean reads were obtained by removing reads containing adapters or ploy-N and low quality reads from raw data by in-houseperl scripts. At the same time, Q20, Q30, GC-content and sequence duplication level of the clean data were calculated. All the downstream analyses were based on clean data with high quality. Transcriptome assembly was accomplished using Trinity [54] with min_kmer_cov set to 2 by default and all other parameters set default. Gene function was annotated based on the plant protein dataset of Nr, Nt, Swiss-Prot database and KOG/COG, respectively, with a significance threshold of *E*-value ≤ 10^−5^. The GO terms for functional categorization were analyzed using Blast2go software with *E*-value ≤ 10^−6^. The pathway assignments were carried out by sequence searches against the KEGG database, using KAAS software with an *E*-value threshold ≤ 10^−10^.

### Differential gene expression analysis

Gene expression levels were estimated by RSEM for each sample [55]. Clean reads were mapped back onto the assembled transcriptome, and read count for each gene was obtained from the mapping results and normalized to FPKM (expected number of fragments per kilobase of transcript sequence per millions base pairs sequenced). Differential expression analysis of two groups was performed using the DESeq R package (1.10.1). The resulting *P*-values were adjusted using the Benjamini and Hochberg’s approach for controlling the false discovery rate. Genes with an adjusted *P*-value <0.05 found by DESeq were assigned as differentially expressed.

Differentially expressed genes (DEGs) were analyzed by GO and KEGG enrichment analysis. GO enrichment analysis was implemented by the GOseq R packages based Wallenius non-central hyper-geometric distribution [56]. The statistical enrichment of DEGs in KEGG pathways were tested by KOBAS [57] software.

### qRT-PCR validation of RNA-Seq expression patterns

Primers to amplify candidate contigs were designed based on sequence of the identified unigenes and listed in Additional file 8. Amplification specificity was checked by comparing the sequences of the amplicon with that of unigene. The 18 s rRNA and ALS were used as internal control genes in qRT-PCR.

Total RNA was extracted and purified using the RNApre Pure Plant Kit (Tiangen, Beijing, China). 1 μg of RNA was used for first-stand cDNA synthesis using the FastQuant RT Kit (Tiangen, Beijing, China). qRT-PCR was conducted in 96-well plates on the ABI 7500 real time PCR system (ABI Life Technologies) using SuperReal PreMix Plus (SYBR Green) (Tiangen, Beijing, China). Reactions were conducted in a 20 μL volume with four replicates for each cDNA sample. Each reaction mixture included 10 μL 2 × SuperReal PreMix Plus, 1 μL diluted cDNA, 0.6 μL primers, 0.4 μL 50 × ROX reference dye, and 7.4 μL RNase-free ddH_2_O. qRT-PCR programs consisted of 15 min incubation at 95 °C, 40 cycles of 95 °C for 10 s, 56 °C for 20 s and 72 °C for 32 s. At the end of the amplification cycle, a melting analysis was carried out to verify the absence of non-specific amplification. Similar amplification efficacy of the target and internal control genes (86.0 %-99.8 %) were observed. Fold-change in gene expression (as 2^-△Ct^) was calculated by the comparative C_T_ method [27], relative to the susceptible samples, where △C_T_ = [C_T_ target gene-C_T_ mean of two internal control genes].

## Abbreviations

ACCase, acetyl-CoA carboxylase; ALS, acetolactate synthase; BP, biologic process; CC, cellular component; DEGs, differentially expressed genes; EPSPS, 5-enolpyruvlshikimate-3-phosphate synthase; FPKM, expected number of fragments per kilobase of transcript sequence per millions base pairs sequenced; GO, gene ontology; GST, glutathione S-transferase; GT, glycosyltransferase; HPPD, 4-hydroxyphenpyruvate dioxygenase; KEGG, Kyoto encyclopedia of genes and genomes; KOG/COG, clusters of orthologous groups of proteins; MF, molecular function; NCBI, National Center for Biotecnology Information; Nr, NCBI non-redundant protein sequences database; Nt, NCBI non-redundant nucleotide sequences database; NTSR, non-target-site based resistance; Pfam, protein family; POD, peroxidase; PPO, protoporphyrinogen IX oxidase; RF, resistance factor; RNA-Seq, high-throughput RNA-sequencing; SRA, sequence read archive; TSR: target-based resistance

## Additional files

Additional file 1: Assessment of assembly quality for resistant (N11) and susceptible (SD8) flixweed populations. (PDF 51 kb) Additional file 2: The length distribution of the transcripts and unigenes. The length distribution of the transcripts (red) and unigenes (blue). (PDF 4 kb) Additional file 3: Species that flixweed unigenes were annotated by BLAST search. (PDF 6 kb) Additional file 4: Pearson correlation (R) between susceptible (SD8) and resistant (N11) samples. (PDF 5 kb) Additional file 5: Volcano plot of differentially expression genes (DEGs) between resistant (N11) and susceptible (SD8) populations. Red spots represent up-regulated DEGs and green spots indicate down-regulated DEGs. Those shown in blue are unigenes that did not show obvious changes. (PDF 146 kb) Additional file 6: Histogram of GO classification of the DEGs. The results are summarized in three main GO categories: biological process, cellular component and molecular function. The x-axis indicates the subcategories, and the y-axis indicates the numbers related to the total number of GO terms present; the DEGs numbers that are assigned the same GO terms are indicated at the top of the bars. (PDF 282 kb) Additional file 7: The scatter plots of top 20 pathways by KEGG enrichment. The x-axis indicates the Rich factor of each pathway, and the y-axis indicates the name for each pathway. Color scale indicates the q-value. The size of the spots indicates the numbers of the DEGs in each pathway. (PDF 6 kb) Additional file 8: Primer information for qRT-PCR validation. (XLSX 14 kb)
