# Supplementary material for: Target-site and non-target-site based resistance to the herbicide tribenuron-methyl in flixweed (Descurainia sophia L.)
Source: BMC Genomics. 2016 Aug 5;17:551. doi: 10.1186/s12864-016-2915-8 (PMC4974779; doi:10.1186/s12864-016-2915-8)
Supplement: Additional file 1: — Assessment of assembly quality for resistant (N11) and susceptible (SD8) flixweed populations. (PDF 51 kb) [file 12864_2016_2915_MOESM1_ESM.pdf]

Additional file 1. Assessment of assembly quality for resistant (N11) and susceptible (SD8) flixweed populations

Sample [a, b]	Raw reads	Clean reads	Clean bases	Error (%)	Q20(%) [c]	Q30(%) [c]	GC(%) [c]
N11_4_1	32048064	31425584	3.93G	0.03	96.44	92.83	46.11
N11_4_2	32048064	31425584	3.93G	0.04	94.09	89.14	46.03
N11_5_1	34964339	34205315	4.28G	0.03	96.60	93.15	46.10
N11_5_2	34964339	34205315	4.28G	0.04	93.55	88.28	46.03
SD8_3_1	37214587	36457368	4.56G	0.03	96.11	92.06	46.15
SD8_3_2	37214587	36457368	4.56G	0.04	92.57	86.47	46.07
SD8_4_1	31833770	31286811	3.91G	0.03	95.60	91.15	45.54
SD8_4_2	31833770	31286811	3.91G	0.04	92.65	86.54	45.48
Summary	272121520	266750156	33.36G				

[a] N11_4 and N11_5 are two plants of the N11 flixweed populations; SD8_3 and SD_4 are two plants of the SD8 flixweed accession;

[b] N11_4_1: left reads of sample N11-4; N11_4_2: right reads of sample N11-4;

[c] Q20 and Q30: percentage of bases with a Phred value >20 and >30 respectively.
