# Supplementary figures and images for: Target-site and non-target-site based resistance to the herbicide tribenuron-methyl in flixweed (Descurainia sophia L.)
Source: BMC Genomics. 2016 Aug 5;17:551. doi: 10.1186/s12864-016-2915-8 (PMC4974779; doi:10.1186/s12864-016-2915-8)

# Length Distribution

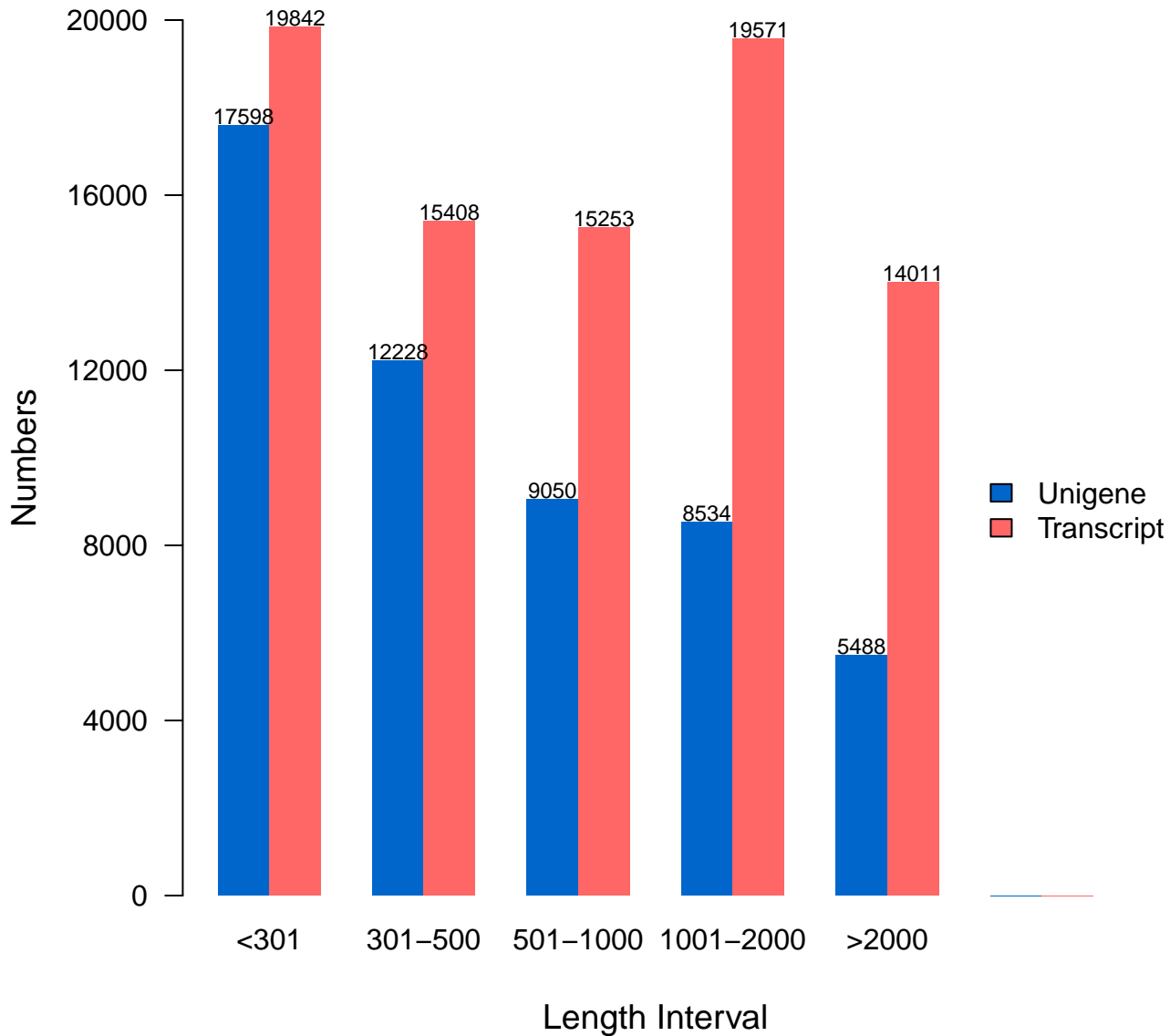

Supplement: Additional file 2: — The length distribution of the transcripts and unigenes. The length distribution of the transcripts (red) and unigenes (blue). (PDF 4 kb) [file 12864_2016_2915_MOESM2_ESM.pdf]

# Pearson correlation between samples

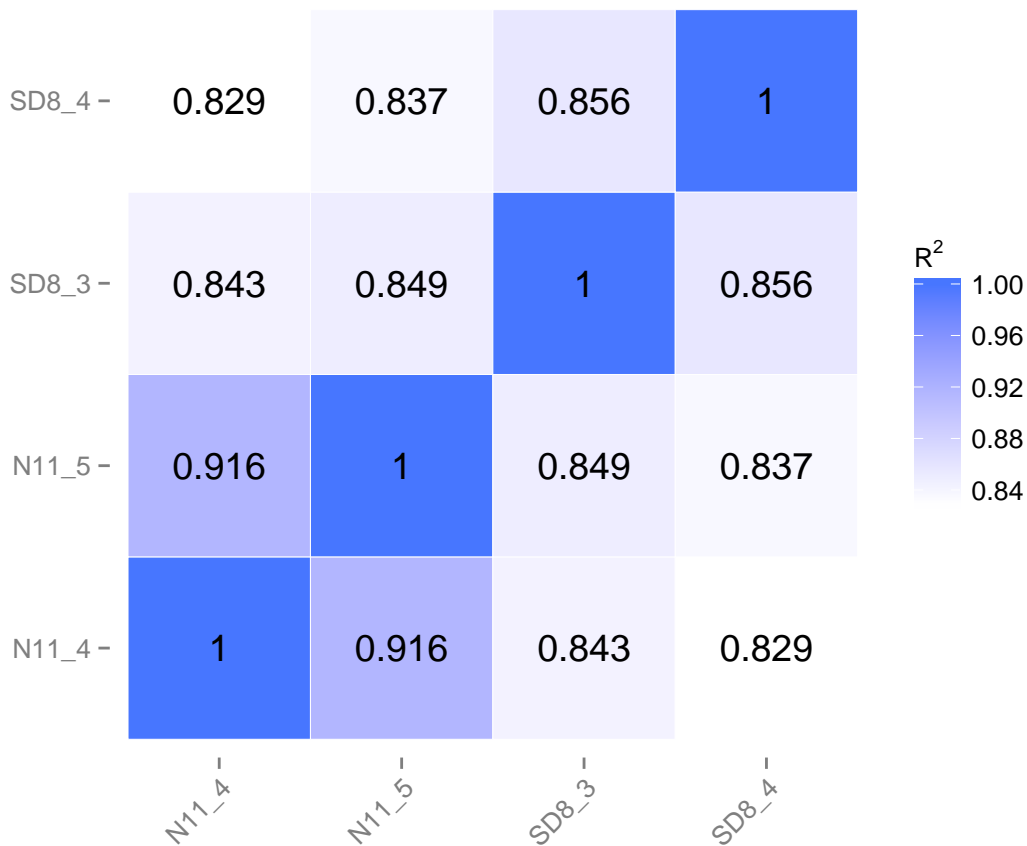

Supplement: Additional file 4: — Pearson correlation (R) between susceptible (SD8) and resistant (N11) samples. (PDF 5 kb) [file 12864_2016_2915_MOESM4_ESM.pdf]

# BNH\_N11 vs BNH\_SD8

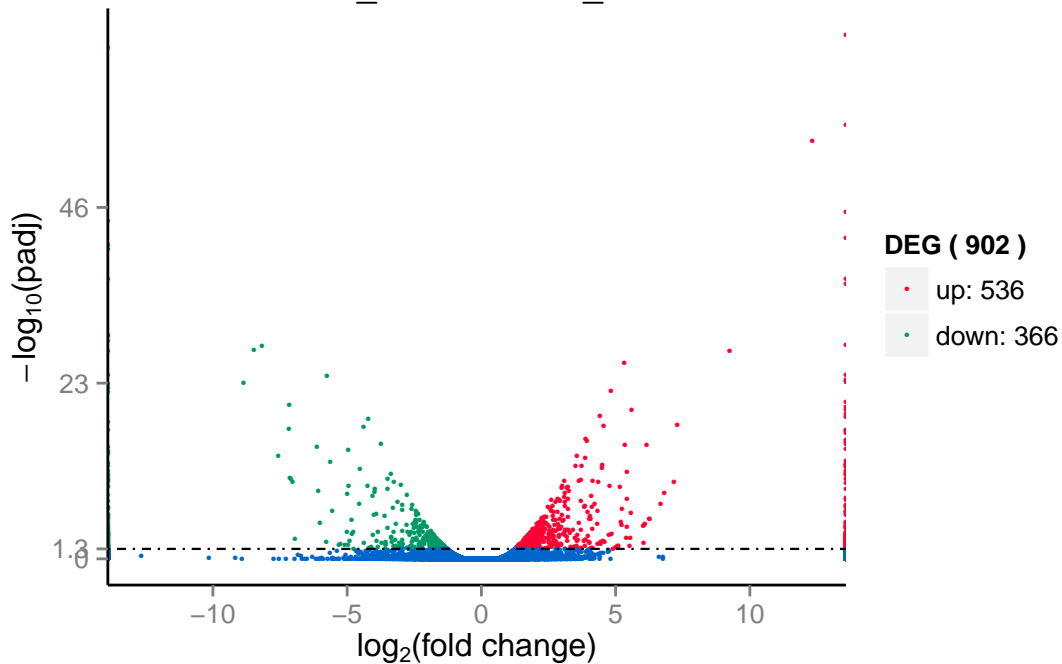

Supplement: Additional file 5: — Volcano plot of differentially expression genes (DEGs) between resistant (N11) and susceptible (SD8) populations. Red spots represent up-regulated DEGs and green spots indicate down-regulated DEGs. Those shown in blue are unigenes that did not show obvious changes. (PDF 146 kb) [file 12864_2016_2915_MOESM5_ESM.pdf]

**A** Enriched GO Terms  
(BNH\_N11vsBNH\_SD8\_up)

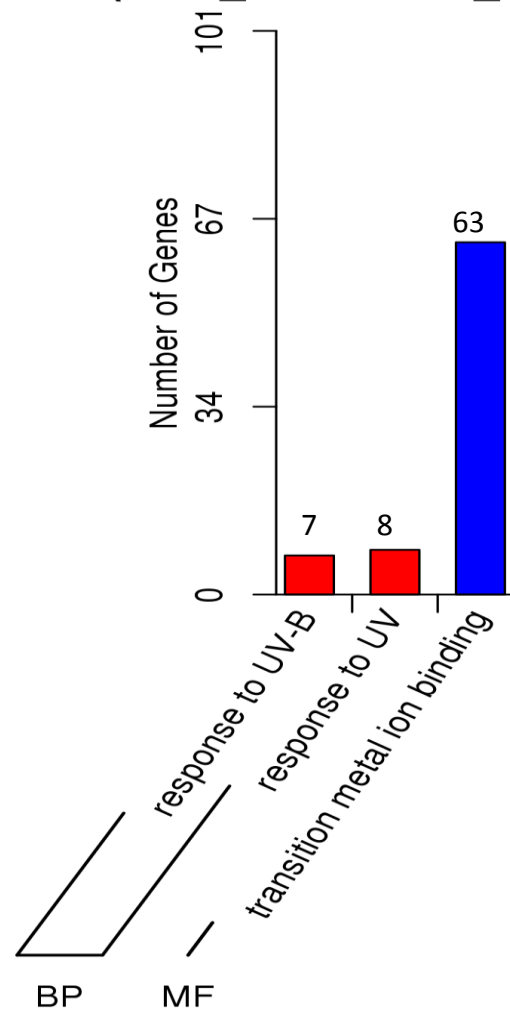

**B** Enriched GO Terms  
(BNH\_N11vsBNH\_SD8\_down)

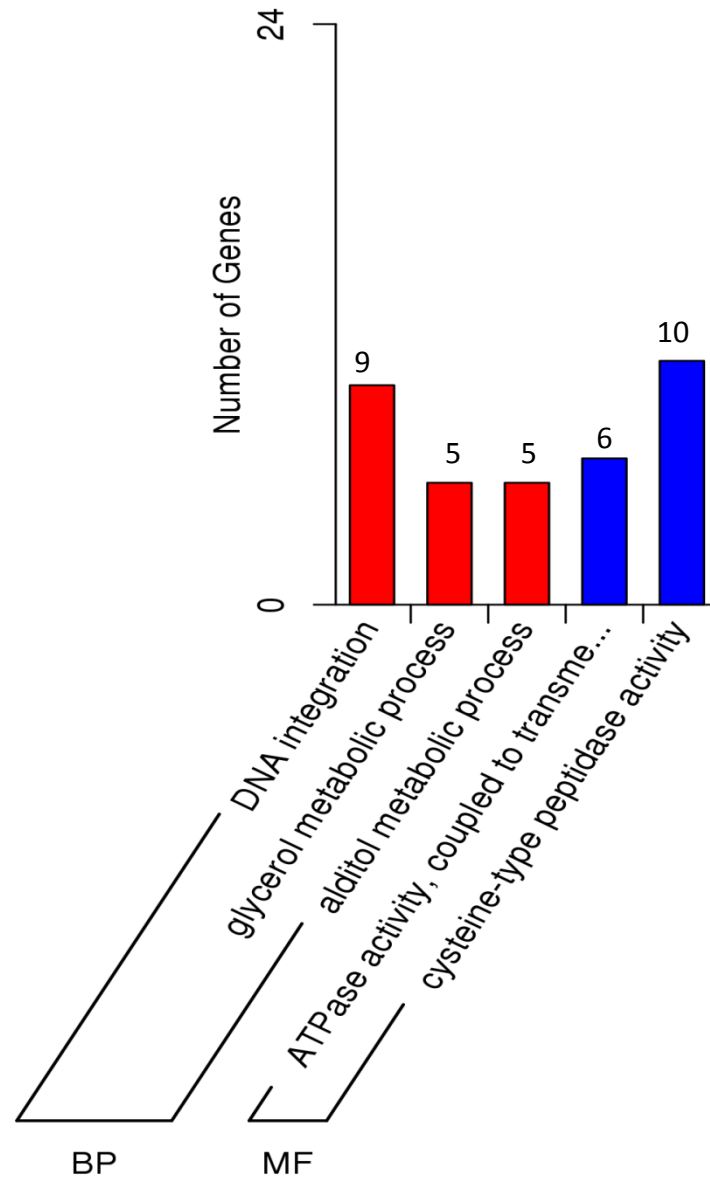

Supplement: Additional file 6: — Histogram of GO classification of the DEGs. The results are summarized in three main GO categories: biological process, cellular component and molecular function. The x-axis indicates the subcategories, and the y-axis indicates the numbers related to the total number of GO terms present; the DEGs numbers that are assigned the same GO terms are indicated at the top of the bars. (PDF 282 kb) [file 12864_2016_2915_MOESM6_ESM.pdf]

# Statistics of Pathway Enrichment

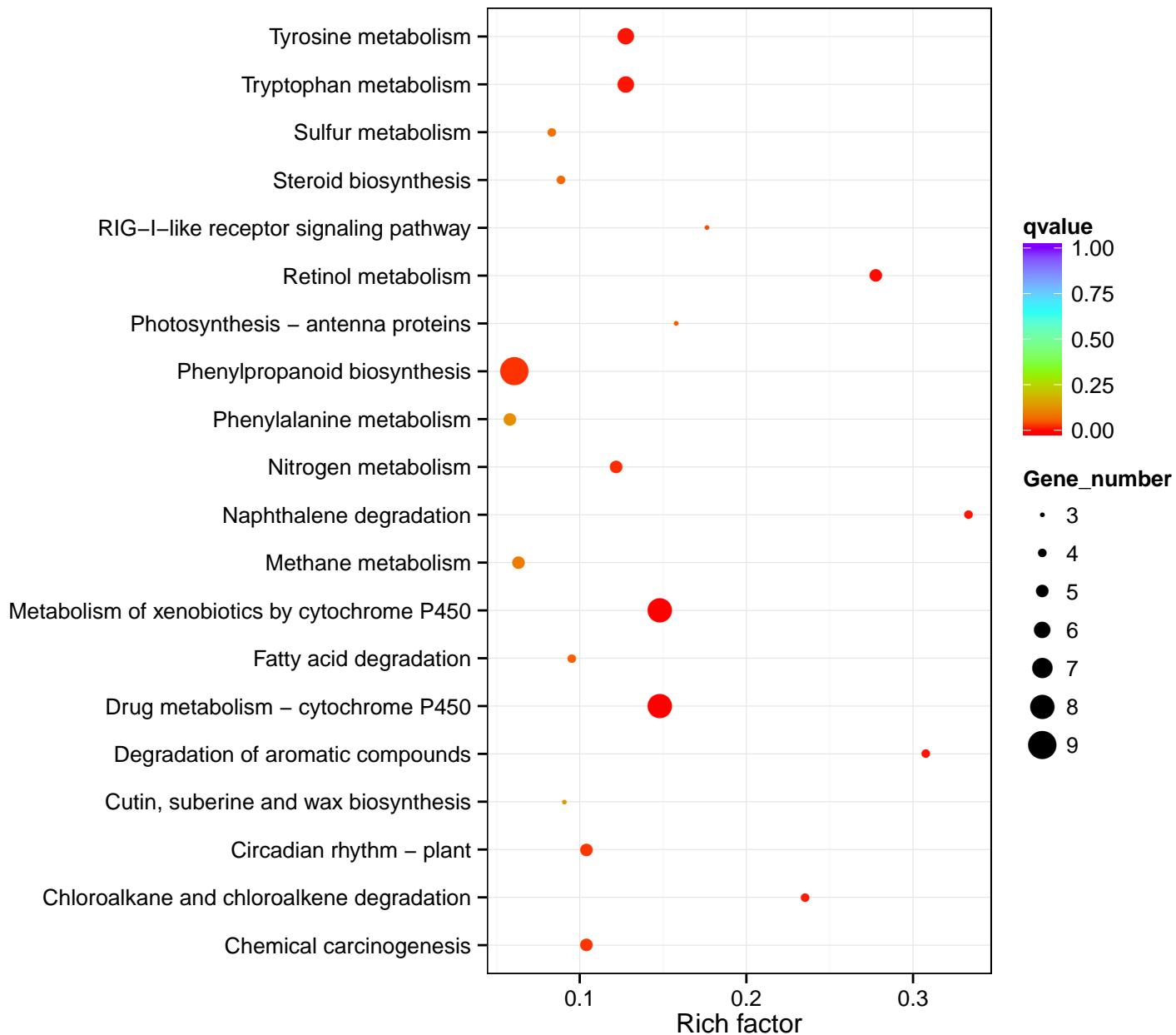

Supplement: Additional file 7: — The scatter plots of top 20 pathways by KEGG enrichment. The x-axis indicates the Rich factor of each pathway, and the y-axis indicates the name for each pathway. Color scale indicates the q-value. The size of the spots indicates the numbers of the DEGs in each pathway. (PDF 6 kb) [file 12864_2016_2915_MOESM7_ESM.pdf]
