# Supplementary material for: Target-site and non-target-site based resistance to the herbicide tribenuron-methyl in flixweed (Descurainia sophia L.)
Source: BMC Genomics. 2016 Aug 5;17:551. doi: 10.1186/s12864-016-2915-8 (PMC4974779; doi:10.1186/s12864-016-2915-8)
Supplement: Additional file 3: — Species that flixweed unigenes were annotated by BLAST search. (PDF 6 kb) [file 12864_2016_2915_MOESM3_ESM.pdf]

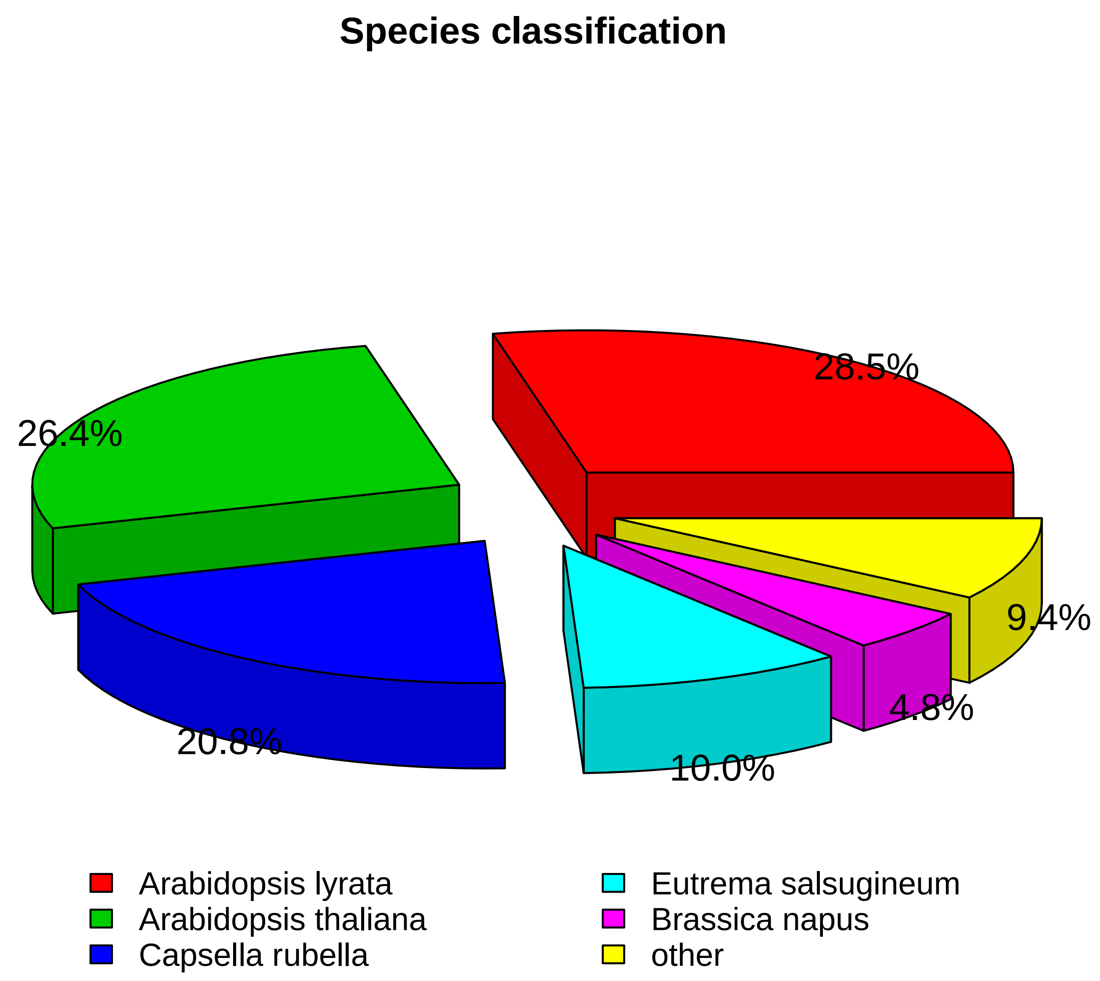
Species classification
28.5%
26.4%
20.8%
10.0%
4.8%
9.4%
Arabidopsis lyrata
Arabidopsis thaliana
Capsella rubella
Eutrema salsugineum
Brassica napus
other
